# Application of HPTLC with Densitometry for Evaluation of the Impact of External Factors on Contents of Diphenhydramine in Its Solutions

**DOI:** 10.1155/2017/4914292

**Published:** 2017-09-18

**Authors:** Katarzyna Bober

**Affiliations:** Department of Analytical Chemistry, School of Pharmacy with the Division of Laboratory Medicine, Medical University of Silesia in Katowice, Jagiellońska 4, 41-200 Sosnowiec, Poland

## Abstract

The subject of durability of drugs is a very important problem investigated by researches. The method of accelerated aging is very often used for stability testing. It involves the influence of temperature, humidity, and light exposure. The aim of this work is estimation of contents of diphenhydramine in its standard solutions undergoing the impact of external factors, that is, temperature and UV light. The standard solutions of diphenhydramine were prepared in distilled water. The analysis of contents of compound investigated was carried out by use of HPTLC. Adsorption thin layer chromatography was performed on aluminum HPTLC plates precoated with silica gel 60F_254_ using mixture of ammonia, methanol, and ethyl acetate as mobile phase. The solutions investigated were exposed to UV light as well as being heated in 40°C. The contents of diphenhydramine were measured in initial solution and during experiment. The additional peaks on densitograms as well as changes in color of solution were observed as a result of exposure to UV light which can give information about new substances, degradation products of diphenhydramine, which were created during experiment.

## 1. Introduction

**Figure 1:**
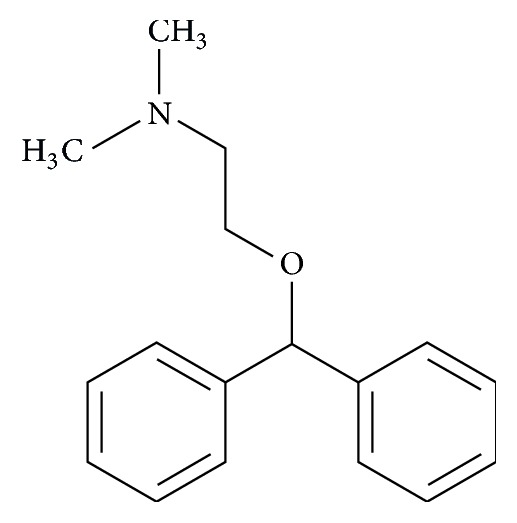
Structural formula of diphenhydramine.

Diphenhydramine is a derivative of arylalkylamine. Its structural formula is presented in Figure 1.

Diphenhydramine is a biological active compound with antihistamine, analgesic, and sedative properties. It is component of many pharmaceutical preparations where it is present in form of hydrochloride [1, 2]. There are many different methods of analysis of diphenhydramine. Usually the method of liquid chromatography combined with mass spectrometry is applied [3–5] as well as liquid chromatography itself [6–14]. Some of the researches use also capillary electrophoresis [15, 16]. There are also some papers describing the thin layer chromatography application to determine the diphenhydramine [17–19] but they do not take into consideration the stability of this biological active substance. Many considerations concern the spectrophotometric analysis of diphenhydramine as well [20–24]. Research concerning the products of degradation of diphenhydramine was also undertaken. The method of SPME-GC/MS was used for the identification of the products of diphenhydramine hydrolysis [25]. The degradation products such as benzhydrol, benzophenone, and dimethylaminoethanol were detected. Degradation was also carried out by using active TiO_2_ photo catalysis [26] but in that case no identification of degradation product was carried out. The aim of this work was estimation of contents of diphenhydramine in its solutions undergoing the impact of external factors, that is, temperature and UV light. It seems that TLC method of analysis of diphenhydramine is the easiest way for this purpose. That is why this method of analysis combined with densitometry is chosen in the work. Thin layer chromatography is also successfully used for analysis of many classes of compounds used in our previous works [27–30].

The drugs stability is a very important problem investigated by researches because of degradation products that are formed and can cause risks for patients [31]. The changes that are observed as a results of chemical, physical, or biological processes can appear during drugs storage [32]. Very often the method of accelerated aging is used for stability testing. It involves the influence of temperature as well as the humidity and light exposure [31, 32]. The influence of humidity is mostly important for solid drug. The accelerating aging tests used by scientists shorten the time of analysis [32].

The aim of this work is to apply thin layer chromatography combined with densitometry to examine the contents of diphenhydramine during heating and exposure to UV light.

## 2. Materials and Methods

### 2.1. Thin Layer Chromatography

The standard solutions of diphenhydramine (Sigma-Aldrich, USA) were prepared in distilled water in concentration of 5 mg/mL. Adsorption thin layer chromatography was performed on aluminum HPTLC plates precoated with silica gel 60F_254_ (#05548 Merck, Germany). The mixture of ammonia (POCh, Poland), methanol (POCh, Poland), and ethyl acetate (POCh, Poland) in volume ratio 2.5 : 5 : 42.5 was used as a mobile phase. Chemicals used as components of mobile phase were of analytical grade. Chromatographic plates used were previously activated during 30 minutes in temperature of 120°C. Solution of diphenhydramine was spotted in amount of 5 *μ*L by use of micro capillary (Camag, Switzerland). Plates were developed in glass chamber, previously saturated during 30 minutes with vapor of mobile phase. Plates were developed to the height of 7.5 cm in a room temperature. The dilutions of standard solution were also prepared for the purpose of getting the standard curve of diphenhydramine determination. The exposure to UV light was used for the purpose of the visualization of chromatographic spots.

### 2.2. Spectrodensitometric Analysis

The spectrum was performed using Camag Scanner TLC3. The radiation sources were deuterium and wolfram lamps. The start wavelength was 200 nm and the end wavelength was 400 nm. The slit dimensions were 8.00 × 0.40 nm; the scanning speed was 100 nm/s. The measurement mode was absorption. Densitometric scanning was then performed with a Camag Scanner TLC3 controlled by winCATS 1.4.1 software. The contents of diphenhydramine in solutions were calculated using previously settled correlation equations on the base of values of height and area of densitometric peaks obtained.

### 2.3. Method Validation

Method validation was carried out according to ICH Guideline [33, 34]. The linearity, limit of detection (LOD), and limit of quantification (LOQ) as well as precision and accuracy were determined for method proposed. Linearity was determined by preparing dilution of standard solution of diphenhydramine. Series of 15 solutions were obtained, which contained, respectively, 15.00, 9.00, 5.40, 3.25, 1.95, 1.15, 0.70, 0.40, 0.25, 0.15, 0.10, 0.05, 0.03, 0.02, and 0.01 mg of diphenhydramine in 5 mL of solution. Solution was spotted in amount of 5 *μ*L onto plate and the plate was developed using mixture of ammonia, methanol, and ethyl acetate in volume ratio 2.5 : 5 : 42.5 as mobile phase. The analysis was performed three times. The calibration curve was then prepared as a relationship between peak area [AU] and contents of diphenhydramine [*μ*g/spot].

Limit of detection (LOD) and limit of quantification (LOQ) were determined on the basis of standard curve obtained using the following equations:

(1) LOD=3.3σSLOD=10σS,

where *σ* is the standard deviation of the response; *S* is the slope of the calibration curve.

Accuracy was evaluated on the basis of value of recovery. Percent of recovery was performed by standard addition method. The known amount of diphenhydramine standard was added to the sample for that purpose, in quantities of 80%, 100%, and 120% of concentration level. The analysis was carried out six times. The percentage of recovery for compound investigated was calculated. Precision was determined on the basis of densitometric measurements of obtained spots as a relative standard deviation, RSD [%].

### 2.4. Accelerating Aging of Diphenhydramine Solutions

The solutions investigated were directly exposed to UV light and separate solutions of diphenhydramine heated in 40°C for the purpose of accelerating aging. The densitometric measurements of contents of diphenhydramine in solutions investigated were done after 10, 20, 30, 50, 70, 90, and 120 hours of exposure to light and heating.

## 3. Results and Discussion

The correlation curves were obtained and described by mathematical equations on the basis of values of particular densitometric peaks area. Parameters concerning the regression equation obtained as well as parameters of method validation are presented in Table 1.

Spectrodensitometric analysis showed that the maximum wavelength for diphenhydramine determination is 200 nm. This wavelength was used for subsequent analysis.

**Figure 2:**
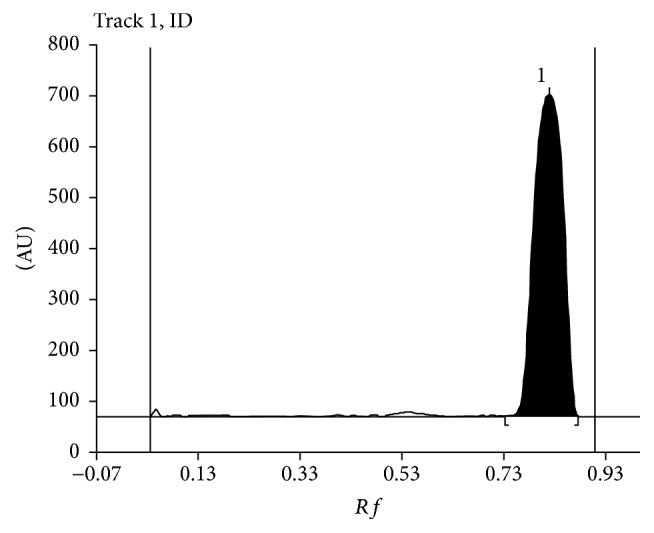
Densitogram of initial solution of diphenhydramine, *λ*_max_ = 200 nm; *R*_*F*_ = 0.82.

Figure 2 presents densitogram of initial solution of diphenhydramine.

**Figure 3:**
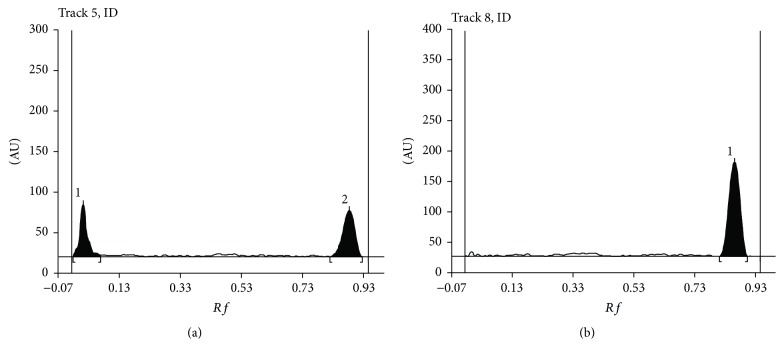
Densitograms of diphenhydramine after 50 h of exposure to UV light (a) and after 50 h of heating (b), *λ*_max_ = 200 nm; *R*_*F*_ = 0.82 (a), *R*_*F*_ = 0.81 (b).

Figures 3(a) and 3(b) present densitograms of diphenhydramine after 50 h of exposure to UV light (Figure 3(a)) and after 50 h of heating (Figure 3(b)). Conditions of densitometric analysis were as in the case of initial solution.

**Figure 4:**
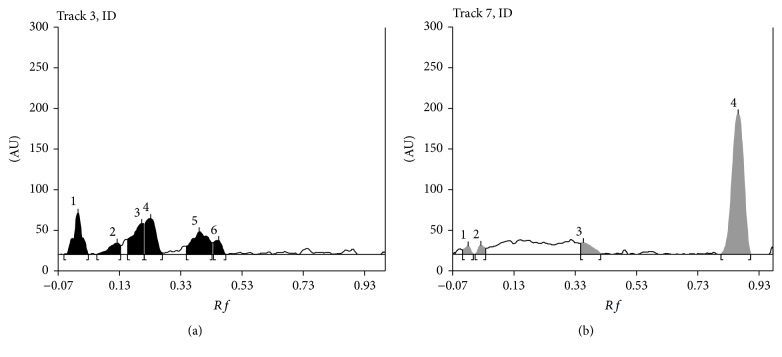
Densitograms of diphenhydramine after 120 h of exposure to UV light (a) and after 120 h of heating (b), *λ*_max_ = 200 nm; *R*_*F*_ = 0.88 (b).

Figures 4(a) and 4(b) present densitograms of diphenhydramine after 120 h of exposure to UV light (Figure 4(a)) and after 120 h of heating (Figure 4(b)). Conditions of densitometric analysis were as in the case of initial solution.

**Figure 5:**
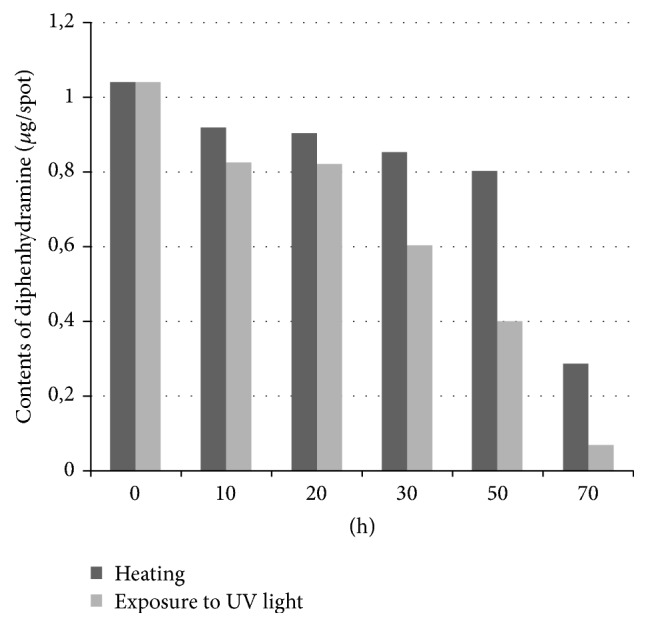
Changes of diphenhydramine contents in time during heating and exposure to UV light.

The contents of diphenhydramine during experiment were calculated for solution heated and exposure to UV light, respectively, on the basis of previously settled calibration equation.  Figure 5 presents the changes in content of diphenhydramine in time during heating and exposure to UV light.

Contents of diphenhydramine were getting smaller and smaller during the experiment. However the changes of diphenhydramine contents during exposure to UV light were more visible and clearly, and at the end of experiment, reached almost zero. Also the additional peaks on the densitogram appeared as a result of exposure to UV light. In the case of heating only small peaks appeared at the end of experiment. The new peaks on the densitogram can be the products of degradation of diphenhydramine. It was also noticed that already after 90 minutes the change of color (into yellow) of solutions exposed to UV light took place. Taking into consideration that solutions were prepared from standard substance characterized by high level of purity (≥98%), it can be stated that the additional peaks visible in the chromatogram of solution investigated are results of exposure to UV light and they are products of diphenhydramine degradation. Pastrana-Martinez and others [26, 35] worked on the degradation of the diphenhydramine using photocatalysts such as TiO_2_ [26] and reduced graphene oxide-TiO_2_ composite [35]. In the first case no product of degradation was identified but the presence of reactive radicals (hydroxyl and hydroperoxyl) was stated. In the second case almost complete degradation was observed after experiment but also no degradation product was identified. In both cases the mechanism of degradation with use of catalysts was analyzed. The method of heating and exposure to UV light without any catalyst and the method of diphenhydramine analysis used in this work (TLC-densitometry) are much easier and faster ways to observe the problem of degradation of biologically active substance investigated. It is noteworthy that analysis of solid substance that underwent the exposure to UV light and heat did not show any changes of diphenhydramine contents, opposite to water solutions of this compound.

Some of the degradation products, as in the case, for example, of hydrolysis degradation products of diphenhydramine [25], can cause that pharmaceutical preparation to be harmful and dangerous for patients.

## 4. Conclusion

The experiment showed that more visible changes of diphenhydramine contents gave the exposure to UV light. Moreover the additional peaks on the densitogram as well as the change in solution color were observed. The results obtained give some suggestion concerning the storage of diphenhydramine as a standard solution as well as the pharmaceutical preparation containing diphenhydramine as active substance.

## Figures and Tables

**Table 1:** Validation parameters describing the diphenhydramine analysis method.

Linearity range [*μ*g/spot]	0.15 ÷ 1.94	LOD [*μ*g/spot]		0.12

Intercept	235.35 (±93.54)	LOQ [*μ*g/spot]		0.35

Slope	4099.28 (±94.39)		Accuracy (% of recovery)	Precision, RSD [%]

Standard deviation *σ*	143.59	**80%**	106.7	1.77

Determination coefficient *R*^2^	99.79%	**100%**	108.0	1.58

Significance of regression *F*	2937	**120%**	107.3	1.45
